# Frequency of Hypocalcaemia following Total Thyroidectomy

**DOI:** 10.12669/pjms.35.1.93

**Published:** 2019

**Authors:** Naseem Baloch, Saima Taj, Mariyah Anwer, Masooma Naseem

**Affiliations:** 1 Dr. Naseem Baloch, FCPS. Department of Surgery, Jinnah Postgraduate Medical Centre, Karachi, Pakistan; 2 Dr. Saima Taj, FCPS, MRCS. Department of Surgery, Sindh Govt: Korangi Hospital, Karachi, Pakistan; 3 Dr. Mariyah Anwer, FCPS. Department of Surgery, Jinnah Postgraduate Medical Centre, Karachi, Pakistan; 4 Dr. Masooma Naseem, MBBS Student, Ziauddin University, Karachi, Pakistan

**Keywords:** Hypocalcaemia, Total thyroidectomy

## Abstract

**Background & Objective:**

Patients who undergo Total thyroidectomy are at higher risk for postoperative hypocalcaemia, which can lead to significant short and long term morbidity. The aim of this study was to determine the frequency of postoperative hypocalcaemia undergoing Total thyroidectomy.

**Methods:**

A total of 854 patients who underwent Total thyroidectomy and completion thyroidectomy between January 2003 to December 2016 at Endocrine Surgical unit, Jinnah Postgraduate Medical Centre, Karachi, were included in this retrospective study. Data were obtained for demographics, preoperative diagnosis, postoperative calcium levels, extent of surgery and final surgical pathology.

**Results:**

A total of 854 patients underwent Total thyroidectomy. Of these 87.58% (n=748) were malignant and 12.41% (n=106) were benign. Among the malignant and benign patients, 47.3% (n=404) underwent Total thyroidectomy and 52.69% (n=450) underwent completion thyroidectomy. Overall incidence of transient hypocalcaemia was 7% (n=60) and that of permanent hypocalcaemia was 0.11% (n= 1).

**Conclusion:**

Hypocalcaemia is one of major concern following total- thyroidectomy. Meticulous surgical techniques, identification and preservation of vascularity of parathyroid glands are essential in preventing postoperative hypocalcaemia following total thyroidectomy.

## INTRODUCTION

The most common complication following total thyroidectomy is hypocalcaemia, which can be either transient or permanent. The reported incidence of transient hypocalcaemia ranges from 1% to 68%, and that of permanent hypocalcaemia ranges from 0% to 13%.^1^-^5^ Moreover, approximately 20% of transient hypocalcaemia occurs from total thyroidectomy only, it increases to 50% to 60%with bilateral central neck dissection.^6^

The patients with symptomatic hypocalcamia is extremely unpleasant, it prolongs hospital stay. The symptoms includes cramps, tingling sensation, paresthesia, tetanic contractions, seizures, muscle spasms, and prolong QT interval prolongation on electrocardiogram.^7^ The most common cause of postthyroidectomy is injury to parathyroid gland. This may be due to parathyroid devascularisation, obstruction of venous drainage or inadvertent excision. Other mechanisms suggested for posththyroidectomy hypocalcaemia comprises hungry bone syndrome in which there is rapid transfer of calcium into bones following surgical treatment of patients with preoperative thyrotoxicosis and intraoperative haemodilution.^4^,^8^

Currently studies are focusing on search for reliable early predictors of postoperative hypocalcaemia. Monitoring the level of serum total calcium (Ca) or ionized calcium (iCa++), circulating slopes in the change of Ca or iCa ++, measuring intraoperative or standard intact parathyroid hormone level, and making a new algorithm by combining more than two of these values have all been reported as useful predictors.^5^ However, these predictors also facilitate the thyroid surgeon, which patient can be discharge early and hospital budget can be saved.^9^ Our objective was to determine the frequency of postoperative hypocalcaemia undergoing Total thyroidectomy.

## METHODS

A retrospective study was conducted at Endocrine Surgery unit, JPMC Karachi. We analyzed 854 consecutive medical records of patients with benign and malignant thyroid disease who underwent Total thyroidectomy between January 2003 and December 2016. Patients who had concomitant central neck lymph node dissection, patients who had prior or concomitant paratrhyroidectomy, known hyperparathyroidism, preoperative hypocalcaemia and history of previous head and neck related chemotherapy and radiotherapy were excluded from this study. The outcome studied was post-thyroidectomy hypocalcaemia 1^st^, 2^nd^ and 5^th^postoperative period and at six months following surgery.

The postoperative serum calcium was determined on 1^st^ postoperative day, 2^nd^ and 5^th^ postoperative days. There was no standard protocol on measuring serum calcium after1^st^ postoperative day in normocalcaemic patients. Transient hypocalcaemia was defined as serum calcium less than 8.0 mg/dl (2mmol/L) on at least two consecutive measurements or signs and symptoms of hypocalcaemia (perioral numbness, digital paresthesia, or positive Trousseau’s sign). Permanent hypocalcaemia was defined as the need for calcium and or vitamin D supplements to maintain normo-calcaemia at six months or more after the date of surgery.^8^ The postoperative PTH was done only in one case of permanent hypocalcaemia due to limited resources. The medical records were reviewed for variables like age, sex, preoperative diagnosis, extent of surgery, postoperative calcium levels, transient and permanent hypocalcaemia, histopatological records. Data analysis were performed through SPSS version (20.0). Approval for study was obtained from the hospital Ethics Review Committee of Jinnah Postgraduate Medical Centre Karachi.

## RESULTS

In this retrospective study, a total of 854 patients medical records were analyzed. There were 670 female (78.45%) and 184(21.54%) were male patients. The mean age was 42.1 years (range 14-76 years). Among the patients included, 47.3% (n=404) underwent Total thyroidectomy and 52.69%(n=450) underwent completion thyroidectomy. Among these 87.58% (n=748) were malignant and 12.41% (n=106) were benign.

Out of 60 patients who developed hypocalcaemia, 36 (56.6%) were malignant and 26 (43.3%) were benign and one patient who develop permanent hypocalcaemia was from benign group. The highest frequency of transient hypocalcaemia was on 2^nd^ postoperative day that is 3.39% in 29 patients and it was delayed up to 5^th^ postoperative day in 7 (0.81%) patients. There was only one patient 0.11% who required calcium and vitamin D supplement for more than six months postoperatively and follow-up till one year and was considered permanent hypocalcaemia we also did postoperative PTH levels in this patient and that was low.

## DISCUSSION

Post-thyroidectomy hypocalcaemia is a recognized complication with significant short and long term morbidity.^4^,^8^,^10^ It often prolong the hospital stay and significantly increases overall cost of a thyroidectomy and patients discomfort.^11^

**Table-I:** Frequency of Transient and Permanent Hypocalcaemia at different periods.

Overall frequency 7%(n=60)
Transient Hypocalcaemia incidence
1^st^ postoperative day 2.69%(n=23)
2^nd^ postoperative day 3.39%(n=29)
5^th^ postoperative day 0.81%(n=7)
Permanent hypocalcaemia 0.11%(n=1)

Patients who have total thyroidectomy are routinely supplemented with calcium and vitamin D by many surgeons. However, this practice can reduce the number of symptomatic patients, but it can be inconvenient as treatment is expensive and poorly tolerated. It can distort the incidence rate of postoperative hypocalcaemia especially when definitions are based on serum calcium levels.^4^,^12^

The ideal way is to predict which patient will develop hypocalcaemia. By this approach we can only treat patients who truly needs replacement therapy. The intraoperative parathyroid hormone (PTH) assay (quick PTH) has been used as a reliable and rapid method to detect hypoparathyroidisim. However, the high cost of quick PTH has often limited its applicability.^13^,^14^

In our study, we evaluated the serum calcium on 1^st^, 2^nd^ and 5^th^ postoperative day to predict post thyroidectomy hypocalcaemia an alternative to quick PTH level due to limited resources and high cost of PTH. Postoperative hypocalcaemia is frequently observed within two to five days after total or subtotal thyroidectomy. However, in most cases hypocalcaemia reverse spontaneously, but it can remain permanent if it is caused by irreversible injury to parathyroid glands. Permanent hypocalcaemia is a lethal complication which requires lifelong therapy and follow-up

In this retrospective study, postoperative hypocalcaemia was observed in 7% of 854 patients undergoing total and completion thyroidectomy which is lower than Esimontas et al.^1^ who reported 64.2% rate of transient hypocalcaemia in 257 patients. In previous local study transient hypocalcaemia was 21.62% in 74 patients which is higher than our results.^15^ However, different studies used different definitions and are not comparable. Permanent hypocalcaemia was found in only one patient 0.11% which is considerably lower than Edafe O et al.^8^, who reported 5.5% rate of permanent hypocalcaemia in 220 patients.

Following thyroid surgery the range of postoperative hypocalcaemia reported in literature varies widely.^1^ There are various factors which accounts for these differences in literature such as definition of hypocalcaemia, type of thyroid disease, and surgical technique for thyroidectomy.^4^,^8^ Patient who had local thyroidectomy with central lymph node dissection had an increased risk of temporary hypocalcaemia.^4^ However, patients with malignancy who required central lymph node dissection were excluded from our study. In this study 87.58% were operated for malignant and 12.4% were operated for benign thyroid disease. The hypocalcaemia developed in 56.6% of malignant and 43% in benign thyroid disease. Our results are comparable with Kumar et al.^16^

Our policy for total thyroidectomy is to preserve parathyroid glands and its vascularity. We identify parathyroid glands before any dissection for identifying and saving recurrent laryngeal nerve, avoid any electro thermal and ultrasonic device contact near parathyroid glands. We usually start the mobilization of superior pole of thyroid gland from medial to lateral direction with individual ligation of branches from superior thyroid artery, by this maneuver we can save the blood supply of superior parathyroid glands. The liberal resection and autotransplantation of parathyroid glands have a greater risk of transient but significant hypocalcaemia^11^ When the blood supply of parathyroid glands appeared to be compromised it was selectively resected and placed in iced saline parathyroid gland was minced into pieces and auto transplanted in ipsilateral sternocleidomastoid muscle. Number of parathyroid glands or inadvertently excised were not analysed due to small numbers in subgroups. The limitation of our study was frozen section and intraoperative and postoperative PTH was not performed routinely.

## CONCLUSION

Hypocalcaemia is one of the major concern following total thyroidectomy. Meticulous surgical technique, identification and preservation of parathyroid glands and its vascularity is essential in preventing postoperative hypocalcaemia following total thyroidectomy. Postoperative monitoring of serum calcium & early treatment can prevent significant morbidity.

### Authors’ Contribution

**BN:** Conceived & editing of manuscript.

**TS:** Did data analysis, design and manuscript writing.

**BN & AM:** Did review and final approval of manuscript.

**NM:** Did data collection.
